# Clinic Reports

**Published:** 1880-06

**Authors:** L. D. Wilson

**Affiliations:** Clinic of Prof. Maclean


### CLINIC REPORTS.

### *COLLEGE OF MEDICINE AND SURGERY. UNIVERSITY OF MICHIGAN.*

### CLINIC OF PROF. MACLEAN FEBRUARY 11TH.

*Reported by L. D. Wilson.*

### TUMOR OF LOWER JAW.

Mr. R., aged 38 years, appeared at the clinic, to-day, with a tumor on the right side of the lower jaw, the size of a small cocoanut. The tumor began in the submaxillary gland in May last, but troubled him none until in the fall. He could move it freely over the jaw, but it is now firm. It is a malignant glandular tumor, and has continued to grow steadily. There has been supperation in the gland, and the patient has suffered considerably the last two months. His health is otherwise good.

Chloroform being given, the inferior right central incisor was removed, and Prof. Maclean began the operation. He first made an incision along the lower border of the inferior maxillary bone, from the symphasis to the angle, then two incisions at right angles with the first: one from the mental process through the lower lip, and the other along the posterior border of the ramus. Another incision was then made, commencing about the middle of the first one, extending downward and perpendicular with it. The flaps were then dissected back, and the jaw sawed through at the symphasis; the right half of the jaw was then disarticulated and removed with the tumor. The flaps were then brought together and retained by the figure of 8, and interrupted sutures. The operation caused the loss of no blood, and no important structures were injured, the surgeon having skillfully avoided the internal maxillary artery, facial nerve, etc. Small bits of absorbent cotton were placed inside the ckeek, and the patient recovered somewhat dreamy from the anaesthetic. February 25th all ligatures were remowed, the parts healed, and the patient dismissed cured.

A dense fibro-cartilaginous mass will form in the place of the lost portion of the jaw, on which can be placed an artificial denture.

FEBRUARY 18TH.—REMOVAL OF TUMOR.

The patient was a boy of sixteen years. The tumor was of the adenoid or glandular variety, and lay in the parotid region: was about the size of a man's fist. The patient being anæsthetized, Prof. Maclean very speedily removed the growth. The external jugular vein being well marked, it was easy to avoid it. One large artery was tied, which probably nourished the tumor.

FEBRUARY 28TH.—OPERATION FOR ANCHYLOSIS OF LOWER JAW.

The patient, Mr. H., aged 22, had scarlet fever twelve years ago. An abscess followed near the articulation of the right side of the jaw, and pieces of bone exfoliated. This resulted in anchylosis of the joint, and continued to grow worse for eight years The last four years it has remained about the same. He can only open the mouth about a quarter of an inch. The patient being given an anæsthetic, Prof. Maclean made an incision near the angle of the jaw, dissected up the skin and masseter muscle, and then with the saw and bone forceps removed a section of bone about an inch long. Passive movements of the jaw to secure non-union, were ordered.

MARCH 3RD.—EPITHELIOMA.

Mr. B., aged 64, came before the class to-day with an epithelioma of ten years' growth. It is situated above the mastoid process, It has been treated in various ways without relief. Chloride of zinc paste was applied, and the patient given gtt v. of Fowler's solution of arsenic twice a day. March 18th the patient was again before the class, with healthy granulations on the sore and doing well.

MARCH 6TH.—ULCERATION OF NOSE.

Mr. H., aged 35, appeared at the clinic to-day. Six years ago the patient burned the right side of his nose slightly, but not much attention was paid to it at the time. It gradually increased until *Lupus Exedens* formed, and the whole right side of the nose ulcerated and sloughed off. It then gradually healed, leaving the mucuous membrane of the septum exposed.

Chloroform being given, Prof. Maclean operated as follows: An incision was first made from the lower part of the right wing of the nose obliquely upwards and outwards, the flap dissected up and drawn on to the side of the nose and stiched fast. This left a small triangular space, which was closed by stretching the integument and retaining it by sutures. Some smaller incisions were made and the parts brought together. Ten days after another slight incision was made and the flaps readjusted.

MARCH 17TH.—OSTEO SARCOMATUS TUMOR.

Mrs. E., aged 59, appeared at the clinic to-day with a tumor of the superior maxillary bone of the right side. Two years ago she was troubled with neuralgic pains; also, paralysis of the upper and right side of the face. She assigned the cause to her teeth, and had six of them extracted. A short time afterwards the right side of her face began to enlarge, accompanied by a great deal of pain. At times she had difficulty in breathing through the right nostril. The tumor involved the whole of the superior maxillary bone.

The patient being anæsthetized by Chloroform, Prof. Maclean commenced the operation: An incision was first made dividing the upper lip and curving up around the right side of the nose. Another incision was made extending outwards horizontally about half way to the ear. This last incision was about half an inch below the lower margin of the orbit. The bone was then sawed through beneath the malar process, and at the union with its fellow of the opposite side. The muscular attachments being liberated, the whole bone was removed. There was a slight hemorrhage, but it was controlled by ligatures. Lint, soaked in carbolized oil, was then inserted, and the parts brought together and stitched with the interrupted suture and hare-lip needle. The patient has been comfortable since the operation, and the wound healed chiefly by first intention.

